# Correction: Synthesis of vinyl-substituted alcohols using acetylene as a C2 building block

**DOI:** 10.1039/d3sc90023a

**Published:** 2023-02-06

**Authors:** Zhicong Lin, Boxiang Liu, Yu Wang, Siju Li, Shifa Zhu

**Affiliations:** a Key Lab of Functional Molecular Engineering of Guangdong Province, School of Chemistry and Chemical Engineering, South China University of Technology Guangzhou 510640 P. R. China zhusf@scut.edu.cn

## Abstract

Correction for ‘Synthesis of vinyl-substituted alcohols using acetylene as a C2 building block’ by Zhicong Lin *et al.*, *Chem. Sci.*, 2023, https://doi.org/10.1039/d2sc06400f.

The authors regret that work important to establishing the context and history of acetylene chemistry was unfortunately not cited in the original article, which meant that ref. 7 in the original article was incomplete.

The corrected version of ref. 7 can be found below, where ref. 7f, k–n are the newly-added references:

The Royal Society of Chemistry apologises for these errors and any consequent inconvenience to authors and readers.

## Supplementary Material
